# Supplementary material for: Trends in Work Conditions and Associations with Workers’ Health in Recent 15 Years: The Role of Job Automation Probability
Source: Int J Environ Res Public Health. 2020 Jul 30;17(15):5499. doi: 10.3390/ijerph17155499 (PMC7432856; doi:10.3390/ijerph17155499)
Supplement: Supplementary file 1 [file ijerph-17-05499-s001.pdf]

International Journal of
Environmental Research and Public Health

MDPI

Table S1. Description of demographic characteristics, work conditions, and health indicators of workers from 2001 to 2016, stratified by automation probability of jobs. P value for trend analysis tests were shown.

	**2001**	**2004**	**2007**	**2010**	**2013**	**2016**	***p***
Number of workers							
All workers	14691	15288	17042	17263	16530	14948	<0.001
High automation probability	45.64%	44.86%	46.65%	45.08%	38.24%	38.48%	<0.001
Median automation probability	36.26%	35.11%	36.09%	35.89%	43.18%	43.67%	<0.001
Low automation probability	18.10%	20.04%	18.26%	19.02%	18.58%	17.85%	<0.001
Average age							
All workers	38.50±9.04	39.11±9.24	39.48±9.65	40.15±9.86	41.02±10.10	41.84±10.32	<0.001
High automation probability	38.80±9.34	39.30±9.39	39.47±9.90	40.02±10.02	40.10±10.10	40.92±10.13	<0.001
Median automation probability	37.77±8.64	38.73±9.07	39.60±9.45	40.25±9.80	42.46±10.12	43.20±10.50	<0.001
Low automation probability	39.20±8.96	39.34±9.18	39.26±9.40	40.26±9.61	39.54±9.61	40.36±9.89	<0.001
Age>55							
All workers	4.45%	4.44%	5.98%	7.60%	9.66%	11.81%	<0.001
High automation probability	5.21%	4.65%	6.54%	7.68%	8.31%	9.47%	<0.001
Median automation probability	3.25%	4.04%	5.45%	7.47%	11.93%	15.04%	<0.001
Low automation probability	4.96%	4.67%	5.62%	7.64%	7.14%	8.96%	<0.001
Female gender							
All workers	40.75%	41.75%	43.46%	45.03%	44.20%	43.40%	<0.001
High automation probability	57.72%	58.21%	59.29%	60.00%	55.05%	54.09%	<0.001
Median automation probability	20.63%	21.80%	23.87%	26.95%	34.56%	34.16%	<0.001
Low automation probability	38.25%	39.83%	42.58%	43.64%	44.32%	42.95%	<0.001
University or above education							
All workers	16.57%	21.58%	24.08%	30.28%	33.21%	36.11%	<0.001
High automation probability	7.53%	11.11%	14.79%	20.97%	21.63%	25.52%	<0.001
Median automation probability	7.45%	12.00%	14.50%	20.77%	24.87%	29.20%	<0.001
Low automation probability	57.65%	61.80%	66.23%	70.31%	76.48%	75.86%	<0.001
**Work conditions**							
Weekly working hours							
All workers	42.64±9.31	43.38±8.22	43.49±8.15	43.05±8.45	43.07±8.06	42.55±10.89	<0.001
High automation probability	42.95±8.04	43.38±7.15	43.40±7.29	42.78±7.84	43.12±7.24	41.87±8.73	<0.001
Median automation probability	42.98±10.71	44.20±8.96	44.20±8.95	43.70±9.44	43.32±8.89	42.16±10.00	<0.001

Low automation probability	41.09±9.10	41.96±8.84	42.32±8.40	42.47±7.75	42.37±7.55	41.11±9.18	0.436
Long working hours (>48 weekly hours)							
All workers	9.44%	11.20%	12.84%	12.35%	13.21%	10.69%	<0.001
High automation probability	7.02%	8.21%	9.61%	8.94%	10.41%	8.65%	<0.001
Median automation probability	13.27%	15.54%	17.02%	16.58%	15.80%	12.56%	0.243
Low automation probability	7.86%	10.32%	12.63%	12.45%	12.95%	10.52%	<0.001
Shift work							
All workers	14.70%	19.55%	19.36%	22.95%	20.28%	21.46%	<0.001
High automation probability	15.09%	18.51%	18.34%	21.16%	21.08%	21.26%	<0.001
Median automation probability	16.40%	24.27%	21.37%	24.96%	20.64%	21.84%	<0.001
Low automation probability	10.33%	13.62%	15.91%	19.87%	17.79%	20.95%	<0.001
Job demand score							
All workers	-	57.24±15.46	57.70±15.43	-	56.21±17.26	58.38±18.15	0.163
High automation probability	-	55.44±15.66	55.93±15.31	-	54.94±17.12	56.64±17.87	0.672
Median automation probability	-	58.03±14.75	58.71±14.92	-	56.39±17.20	58.39±18.01	0.637
Low automation probability	-	59.87±15.75	60.12±16.19	-	58.39±17.43	62.09±18.53	0.001
Job control score							
All workers	-	51.74±14.56	51.48±14.00	-	49.49±13.73	48.46±14.00	<0.001
High automation probability	-	47.46±13.66	47.28±13.17	-	45.99±12.72	44.94±12.75	<0.001
Median automation probability	-	51.51±13.89	51.77±13.16	-	48.67±13.46	47.69±13.64	<0.001
Low automation probability	-	61.64±12.77	61.41±12.43	-	58.62±12.30	57.98±13.22	<0.001
Job insecurity							
All workers	49.85%	56.05%	53.78%	47.37%	51.82%	47.86%	<0.001
High automation probability	53.04%	60.02%	57.21%	50.37%	57.05%	52.25%	<0.001
Median automation probability	55.27%	62.08%	58.55%	51.71%	54.20%	50.74%	<0.001
Low automation probability	30.96%	36.61%	35.80%	32.06%	35.52%	31.33%	0.399
**Health conditions**							
Poor self-rated health							
All workers	4.60%	3.99%	3.19%	3.21%	3.53%	4.14%	0.005
High automation probability	5.27%	4.13%	3.38%	3.64%	3.36%	4.20%	<0.001
Median automation probability	4.08%	3.80%	3.27%	2.83%	3.99%	4.70%	0.063
Low automation probability	3.97%	4.02%	2.57%	2.90%	2.82%	2.67%	<0.001
Burnout (score>50)							
All workers	-	15.36%	14.08%	-	11.71%	12.42%	<0.001
High automation probability	-	14.84%	13.89%	-	10.72%	11.65%	<0.001
Median automation probability	-	14.83%	13.48%	-	11.98%	12.18%	<0.001
Low automation probability	-	17.43%	15.71%	-	13.11%	14.69%	<0.001
